# Sodium polystyrene sulfonate (Kayexalate) aspiration

**DOI:** 10.1186/1746-1596-3-27

**Published:** 2008-06-17

**Authors:** Luis F Gonzalez-Cuyar, Nathaniel B Cresswell, Allen P Burke

**Affiliations:** 1 Department of Pathology University of Maryland School of Medicine, 22 South Greene Street, NBW64, Baltimore, Maryland, USA

## Abstract

In this short report we illustrate a case of extensive sodium polystyrene sulfonate (SPS) aspiration as an immediate cause of death in a terminally ill patient. SPS is a cation exchange resin utilized to decrease potassium levels in patients with renal failure. When administered rectally in conjunction with sorbitol, colonic necrosis and perforation have been documented. On the other hand, oral administration can be complicated by aspiration, especially in very ill or debilitated patients. In our current report, histological examination of a patient who aspirated SPS shows multiple polygonal to amorphous basophilic crystalline particles deposited intraalveolarly. The purpose of our report is to familiarize pathologists with the histologic features of this rare iatrogenic complication of therapy for hyperkalemia.

## Findings

We report a case of a 45 year-old male patient with a history of HIV, Hepatitis B, and Hepatitis C secondary to illicit drug use. He also suffered from end-stage renal disease with persistent hyperkalemia which was treated with hemodialysis. The patient was admitted for a total right hip arthroplasty as a treatment for severe osteoarthritis. On post operative day four the patient suffered changes in mental status, fever, and decreased saturations and was treated for a presumptive diagnosis of pneumonia. On post operative day nine potassium levels remained elevated and the patient was administered 15 grams of SPS solution orally. One hour later the patient developed poor saturations, was severely hypoxemic, deteriorated, and expired.

Post mortem gross examination revealed severe pulmonary congestion (right lung 1,260 grams; left lung 1,080 grams) with focal consolidations. There was no evidence of emboli and the airways were free of any gastric contents. Additional pertinent gross findings included a remote left ventricular infarct, cardiomegaly (680 grams), and hepatosplenomegaly (liver 2,240 grams; spleen 430 grams).

**Figure 1:**
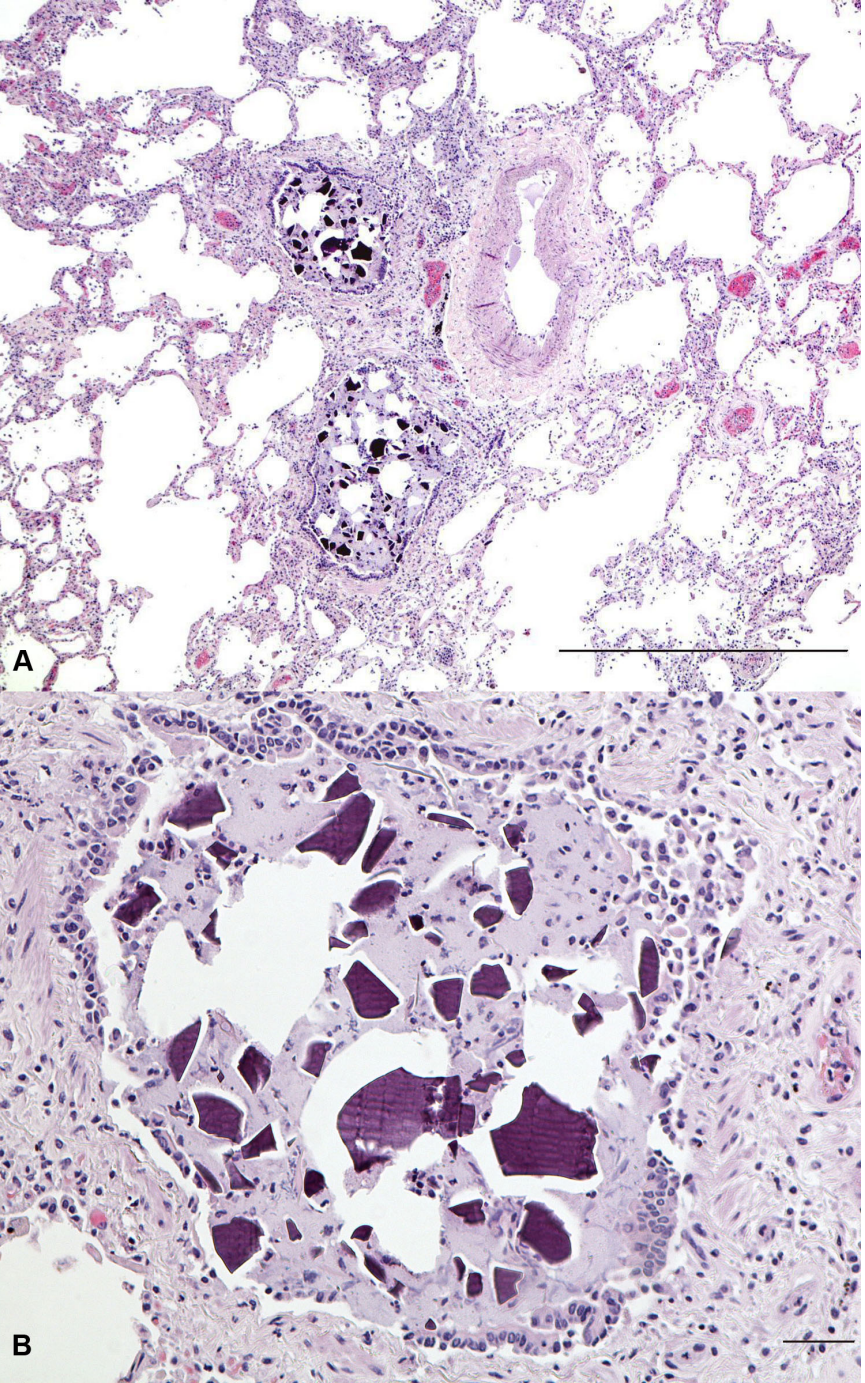
**A – Medium power magnification demonstrating purple polygonal intraalveolar kayexalate crystals on a background of emphysematous changes**. Scale bar 1000 μm. B – High power magnification showing the SDS crystals in high detail measuring up to 60 μm as well as smaller amorphous ones. Scale bar 20 μm.

Histological examination of all lung lobes revealed extensive intraalveolar deposition of purple polygonal to amorphous crystals ranging in measurement from 1 to 60 micrometers (Figure 1). Additionally, there were geographic foci of bacterial bronchopneumonia and diffuse emphysematous changes. Tissue gram stain was positive for gram-positive cocci in chains.

## Discussion

The administration of sodium polystyrene sulfonate (SPS) is used extensively for the treatment of hyperkalemia, especially in patients with renal failure [1-3]. Other uses have been reported, however [4-7]. SPS is an ion-exchange resin that exchanges Na^+ ^for K^+ ^in the gastrointestinal tract, especially in the colon, and increases the fecal excretion of potassium ions. It can be administered orally or rectally suspended in sorbitol, water, or dextrose. Historically, reports of ischemic colitis and colonic necrosis after rectal administration of SPS in sorbitol solution are abundant in the literature, but rare for the upper gastrointestinal tract [8-18]. Other complications such as thrombocytopenia [19] and rectal stenosis [20] have been reported. Administration of SPS suspended in dextrose or water has been suggested to avoid sorbitol related complications; however, recent reports have shown sorbitol-free SPS enemas to cause similar colonic catastrophes [21].

SPS aspiration is a potential complication of oral administration, especially in debilitated patients [22,23]. One study reports an incidence of intestinal necrosis in 1.8% of post operative patients [13]. The aspiration of SPS was first described by Oi [24] in 1978 in a post term hyperkalemic female infant with meconium aspiration. After a second oral administration the infant became cyanotic, bradycardic, apneic, and expired [24]. The microscopic appearance of the intraalveolar material was then compared to a solution of 25% SPS which yielded similar histological features [24]. In an animal model, Haupt et al [25] demonstrated that the inflammatory reaction following SPS aspiration is not solely attributed to concomitant aspiration of gastric contents.

The purpose of this report is to document a rare, often undiagnosed, and fulminant cause of death among patients with renal failure under the administration of potassium lowering agents. The particular appearance of SPS on histological sections should highlight it as a possible cause of aspiration pneumonia. In our patient, SPS aspiration superimposed on an already compromised pulmonary function was the immediate cause of death. Both methods of administration of the cation exchange resin have been proven effective, but rectal administration may prove beneficial in avoiding aspiration pneumonitis in chronically ill and debilitated patients.

## Competing interests

The authors declare that they have no competing interests.

## References

[B1] Gerstman BB, Platt R (1991). Use of sodium polystyrene sulfonate in sorbitol in the United States, 1985–1989. Am J Kidney Dis.

[B2] Osawa A, Okoshi M, Higuchi J, Yamayoshi W (1969). [Treatment of hyperkalemia in renal insufficiency with cation exchange resin. Experience with use of sodium polystyrene sulfonate]. Hinyokika Kiyo.

[B3] Takasu T (1970). [Treatment of hyperkalemia associated with renal insufficiency – clinical effects and side reactions of positive-ion-exchange resins, sodium polystyrene sulfonate (Kayexalate)]. Nippon Rinsho.

[B4] Linakis JG, Hull KM, Lacouture PG, Lockhart GR, Lewander WJ, Maher TJ (1996). Sodium polystyrene sulfonate treatment for lithium toxicity: effects on serum potassium concentrations. Acad Emerg Med.

[B5] Linakis JG, Hull KM, Lacouture PG, Lockhart GR, Lewander WJ, Maher TJ (1997). Enhancement of lithium elimination by multiple-dose sodium polystyrene sulfonate. Acad Emerg Med.

[B6] Linakis JG, Savitt DL, Wu TY, Lockhart GR, Lacouture PG (1998). Use of sodium polystyrene sulfonate for reduction of plasma lithium concentrations after chronic lithium dosing in mice. J Toxicol Clin Toxicol.

[B7] Shepherd G, Klein-Schwartz W, Burstein AH (2000). Efficacy of the cation exchange resin, sodium polystyrene sulfonate, to decrease iron absorption. J Toxicol Clin Toxicol.

[B8] Bennett LN, Myers TF, Lambert GH (1996). Cecal perforation associated with sodium polystyrene sulfonate-sorbitol enemas in a 650 gram infant with hyperkalemia. Am J Perinatol.

[B9] Burnett RJ (1990). Sodium polystyrene-sorbitol enemas. Ann Intern Med.

[B10] Cheng ES, Stringer KM, Pegg SP (2002). Colonic necrosis and perforation following oral sodium polystyrene sulfonate (Resonium A/Kayexalate in a burn patient. Burns.

[B11] Gales MA, Gales BJ, Dyer ME, Orr SR (1995). Rectally administered sodium polystyrene sulfonate. Am J Health Syst Pharm.

[B12] Gardiner GW (1997). Kayexalate (sodium polystyrene sulphonate) in sorbitol associated with intestinal necrosis in uremic patients. Can J Gastroenterol.

[B13] Gerstman BB, Kirkman R, Platt R (1992). Intestinal necrosis associated with postoperative orally administered sodium polystyrene sulfonate in sorbitol. Am J Kidney Dis.

[B14] Marion F, Joye F (1999). [Polystyrene sodium sulfonate enema: with or without sorbitol?]. Presse Med.

[B15] Montagnac R, Mehaut S, Blaison D, Schillinger F (2002). [Colonic necrosis caused by sodium polystyrene (kayexalate) in hemodialysis: myth or reality? Two case reports]. Nephrologie.

[B16] Rashid A, Hamilton SR (1997). Necrosis of the gastrointestinal tract in uremic patients as a result of sodium polystyrene sulfonate (Kayexalate) in sorbitol: an underrecognized condition. Am J Surg Pathol.

[B17] Rogers FB, Li SC (2001). Acute colonic necrosis associated with sodium polystyrene sulfonate (Kayexalate) enemas in a critically ill patient: case report and review of the literature. J Trauma.

[B18] Scott TR, Graham SM, Schweitzer EJ, Bartlett ST (1993). Colonic necrosis following sodium polystyrene sulfonate (Kayexalate)-sorbitol enema in a renal transplant patient. Report of a case and review of the literature. Dis Colon Rectum.

[B19] Mogi Y, Kura T, Takimoto R, Muto F, Maeda T, Muramatsu H, Niitsu Y (1997). [Thrombocytopenia associated with sodium polystyrene sulfonate]. Rinsho Ketsueki.

[B20] Chatelain D, Brevet M, Manaouil D, Yzet T, Regimbeau JM, Sevestre H (2007). Rectal stenosis caused by foreign body reaction to sodium polystyrene sulfonate crystals (Kayexalate). Ann Diagn Pathol.

[B21] Rugolotto S, Gruber M, Solano PD, Chini L, Gobbo S, Pecori S (2007). Necrotizing enterocolitis in a 850 gram infant receiving sorbitol-free sodium polystyrene sulfonate (Kayexalate): clinical and histopathologic findings. J Perinatol.

[B22] Chaplin AJ (1997). Histologic occurrence of polystyrene sulfonates. Arch Pathol Lab Med.

[B23] Idowu MO, Mudge M, Ghatak NR (2005). Kayexalate (sodium polystyrene sulfonate) aspiration. Arch Pathol Lab Med.

[B24] Oi RH (1978). The microscopic appearance of a sodium-potassium exchange resin in histologic sections. Am J Clin Pathol.

[B25] Haupt HM, Hutchins GM (1982). Sodium polystyrene sulfonate pneumonitis. Arch Intern Med.

